# Automated Inflating Resuscitator (AIR): Design and Development of a 3D-Printed Ventilator Prototype and Corresponding Simulation Scenario Based on the Management of a Critical COVID-19 Patient

**DOI:** 10.7759/cureus.9134

**Published:** 2020-07-11

**Authors:** Bruno Gino, Zhujiang Wang, Philip d'Entremont, Tia S Renouf, Adam Dubrowski

**Affiliations:** 1 Emergency Medicine, Madrecor Hospital, Uberlândia, BRA; 2 Pre-Hospital, SIATE - Integrated Trauma and Emergency Assistance System, Uberlândia, BRA; 3 Health Sciences, Ontario Tech University, Oshawa, CAN; 4 General Medicine, University of Limerick, Limerick, IRL; 5 Emergency Medicine, Memorial University of Newfoundland, St. John's, CAN

**Keywords:** healthcare simulation, corona virus, ventilator, engineering, healthcare technology, simulation based medical education, ventilation, covid-19, ppe, 3d printing

## Abstract

Recent surges in COVID-19 cases have generated an urgent global demand for ventilators. This demand has led to the development of numerous low-cost ventilation devices, but there has been less emphasis on training health professionals to use these new devices safely. The aim of this technical report is twofold: first, to describe the design and manufacturing process of the automated inflating resuscitator (AIR), a 3D-printed ventilator training device which operates on the principle of pushing a bag valve mask; second, to present a simulation scenario that can be used for training health professionals how to use this and similar, low-cost, 3D-printed ventilators in the context of ventilator shortages caused by COVID-19. To this end, the AIR was designed in an expedient manner in accordance with basic functionality established by the Medicines and Healthcare Products Regulatory Agency (United Kingdom) for provisional clinical use in light of COVID-19.

## Introduction

In late 2019, a cluster of pneumonia cases with unknown etiology were identified in Wuhan, China. Despite significant containment efforts, COVID-19 has since spread widely. As of June 28, 2020, there have been 495,760 global mortalities, with 8,508 of those coming from Canada [1]. Critical COVID-19 cases are characterized by respiratory failure requiring mechanical ventilation. In some countries, the demand for ventilators has far exceeded existing supplies. In Italy, for instance, physicians have been forced to allocate ventilator access on the basis of triage criteria such as age and likelihood of survival [2]. There is therefore an urgent need for alternative ventilators, which has led to the development of numerous devices. However, there has been less emphasis on how to train health professionals to use these new devices safely. Consequently, in this report we describe the design and development of the automated inflating resuscitator (AIR), a 3D-printed ventilator training device. This technical report begins by considering the design elements that went into the AIR prototype, with reference to guidelines recently established by the Medicines and Healthcare Products Regulatory Agency (MHRA, United Kingdom) [3]. AIR contains the core components of a functional ventilator, with the possibility of further modular augmentation. In addition to the description of the ventilator, we also present a simulation case using AIR to train safety, operational ability, crisis resource management, and communication skills.

## Technical report

Design considerations

The development of the AIR prototype followed MHRA guidelines of the minimally acceptable ventilator to be used in hospitals during the current COVID-19 pandemic [3]. These guidelines influenced the choice of materials, design, and operative functions. This section discusses these considerations by focusing on four areas:

1. bag valve mask 2. oxygen flow 3. pressure valve 4. infection control

Bag valve mask

The bag valve mask (BVM) was chosen for the construction of the AIR as it is inexpensive, easy to use, widely available, and already has its own safety features. BVMs were created to temporarily replace mechanical ventilators during situations where mechanical ventilators are not available, and they function by manually providing positive pressure ventilation for patients who no longer have the ability to breathe [4]. By incorporating the BVM, the AIR is intended to keep patients who need mechanical ventilation on ventilatory support, temporarily stabilizing their condition until a traditional mechanical ventilator becomes available. 

There are two main types of BVM: flow-inflated and self-inflated. Flow-inflated BVMs require a continuous gas flow or must be connected to an electrical source. Self-inflated BVMs expand after the bag is uncompressed. Therefore, a self-inflated BVM, known also under the proprietary name Ambu® (Ambu A/S, Copenhagen, Denmark), was chosen for the AIR because it has this characteristic and because it is portable and easy to use [5].

**Figure 1:**
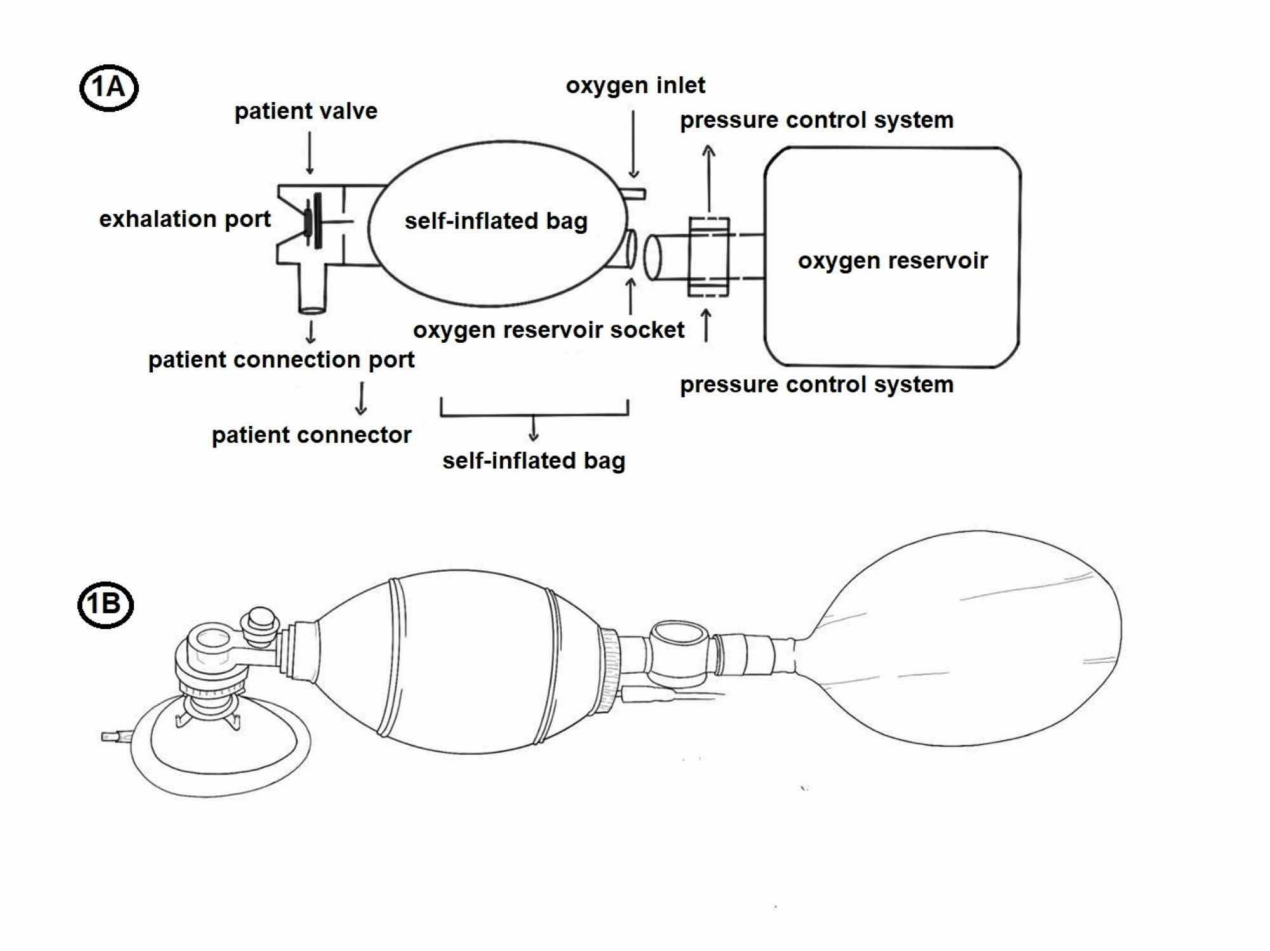
Bag valve mask 1A: Mechanical diagram 1B: Illustration

A BVM has four parts: exhalation port, patient valve, self-inflated bag, and an oxygen inlet. Optional components include a positive end-expiratory pressure (PEEP) valve, an oxygen reservoir, and a pressure gauge [6]. Figure 1A presents a mechanical diagram of BVM components and is intended for engineering purposes. Figure 1B illustrates how these components are assembled on an actual device.

**Figure 2:**
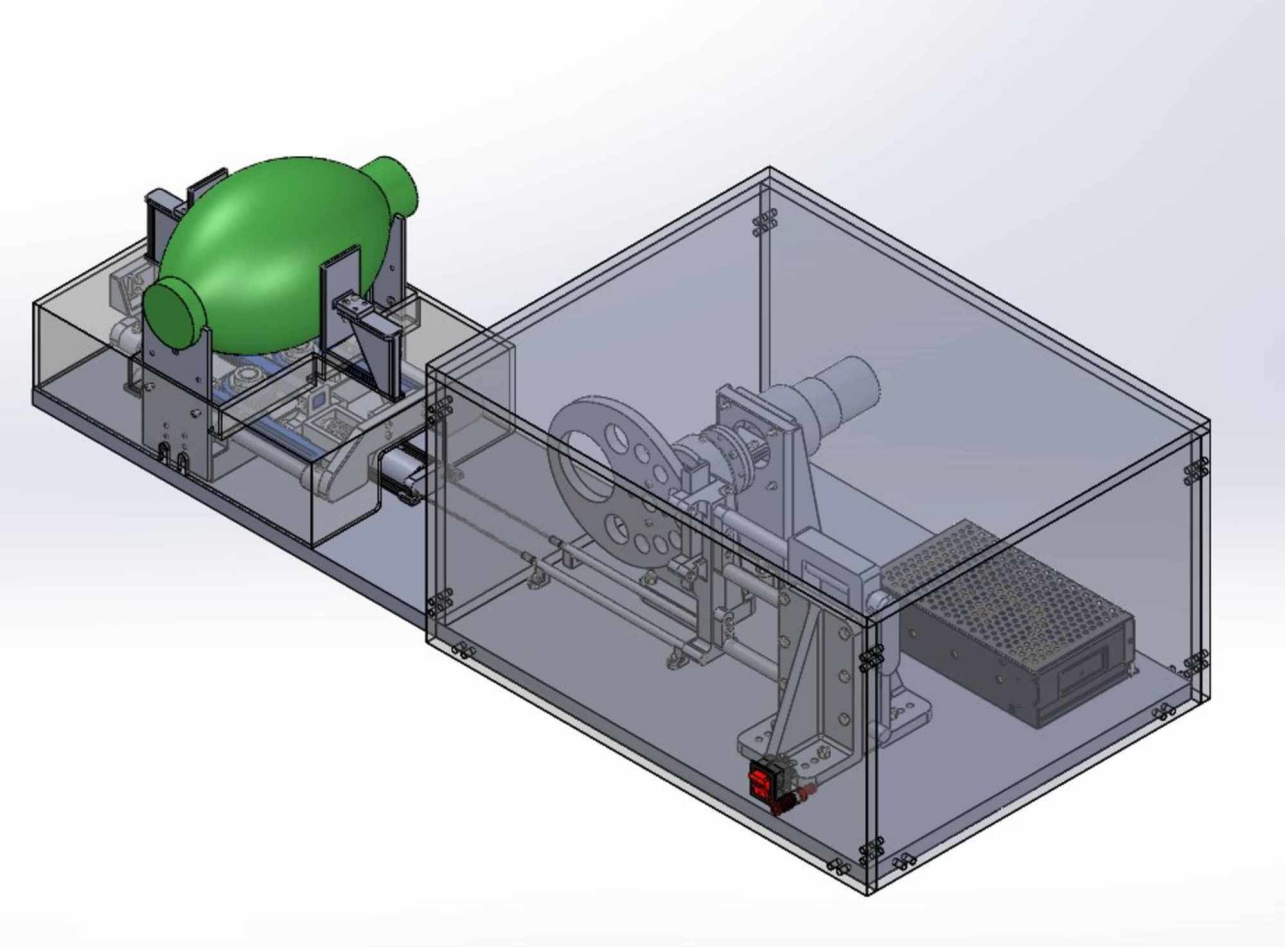
AIR model AIR: automated inflating resuscitator

Each AIR component was purchased, fabricated from commonly available commercial material, or printed using a 3D printer (see Appendix 1). The self-inflated bag is placed separately from electrical components for safety purposes, which also allows it to be easily removed or replaced. The speed of the motor and the position of the bag can be adjusted to control the breath rate and tidal volume (TV) respectively. Solidworks design model and stereolithography (STL) files can be downloaded from GitHub https://github.com/maxSIMhealth/Automated-Inflating-Resuscitator-AIR-. Figure 2 provides a design overview of the AIR model.

AIR parameters are displayed in Table 1. The operator can select their own motor system if the following requirements are met: the rated rotation speed falls in the range of 0-30 rotations per minute (rpm); rated torque is at least 15 Newton centimetres (N⋅cm).

**Table 1:** AIR parameters AIR: automated inflating resuscitator; VCV: volume control ventilation; I:E - inspiratory:expiratory; W: watt; AC: alternating current; V: volt; Hz: hertz; FiO2: fraction of inspired oxygen; TV: tidal volume; PEEP: positive end-expiratory pressure; cmH2O: centimetre of water.

Parameter	Description
Ventilation mode	VCV
I:E ratio	1:2
Respiratory rate	10-30 breaths per minute
Bag volume	1700 ± 100 mL
Maximum power	15 W
Input voltage	AC 110 V 50 Hz
Adjust FiO2	0.21, 0.41, 1.00 (currently only 0.21 or 1.00)
TV	100-900 mL
PEEP	5-60 cmH2O

*Oxygen Flow* The use of an oxygen source connected to the AIR and the location of oxygen entering the balloon can influence the positive expiratory pressure (PEP) and TV generated regardless of the BVM brand [7]. In adults, resuscitators must be able to function properly with high oxygen flows, as during an emergency the flow meter can be changed and the resuscitator must remain in optimal condition. This consideration must be reviewed by all professionals who will eventually use the AIR. A malfunctioning manual or automatic resuscitator can put the patient at risk by providing an inadequate amount of oxygen [8]. There are clinical situations in which professionals must personalize the amount of oxygen and, consequently, the percentage of oxygen mixed with atmospheric air offered to each patient. Some BVMs have been tested using oxygen flows of 1, 5, 10 and 15 litres per minute (L/min) with and without an oxygen reservoir, and this oxygen flow influences the fraction of inspired oxygen (FiO2) offered to the patient [7]. It is therefore important to know how an oxygen reservoir can influence the FiO2 offered by the BVM device. Evidence suggests that a BVM that allows the oxygen reservoir to be coupled, when tested without this accessory, reaches 40% FiO2 when patients receive an oxygen flow greater than or equal to 10 L/min [9]. Therefore, due to the possibility of variation in the performance of the BVM as a function of the oxygen flow used, healthcare professionals must adjust the flow so that the device provides the adequate amount of oxygen for each specific situation. In this case the healthcare professional must choose the specific Venturi valve printed on a 3D printer and connect it to AIR so that FiO2 best adapts to the patient's condition [10]. The principles that govern BVM function also apply to the AIR; therefore, since the former can supply ambient air when no gas source is connected, the latter can be used in situations requiring limited FiO2, such as FiO2 at 21% of the ambient air [9]. When connected to the source of 100% oxygen, it is recommended to use a flow of 5 to 10 L/min at the entrance of the bag. The professional handling the AIR or BVM must also be aware that the position of the oxygen inlet, differences in oxygen flow supplied to the BVM, use of the oxygen reservoir, and the type of oxygen inlet valve can each contribute to FiO2 variability for different resuscitator brands [8]. The professional handling the AIR or BVM should keep in mind that the position of the air inlet, differences in oxygen flow supplied to the BVM, use of the oxygen reservoir, and type of air inlet valve can each contribute to FiO2 variability for different resuscitator brands [8].

Pressure Valves

There are two valves in the reservoir. A safety outlet valve relieves pressure inside the reservoir if excessive airflow is provided or if there is no manipulation of the bag. If the volume of gas in the reservoir is insufficient to fill the bag, another safety inlet valve allows the entry of ambient air. The PEEP valve can be attached to the expiratory port of the AIR. It prevents alveolar derecruitment during manual or automatic ventilation [11].

Another important consideration is the addition of the pressure relief valve to the BVM. In clinical practice health professionals do not usually monitor pressures while using a BVM. Instead they use experiential knowledge based on direct patient observation. These professionals usually adjust the pressure by evaluating the expansion of the rib cage and resistance to ventilation, perceived subjectively by the operator's hands.

However, in an emergency situation, one may not be able to view chest expansion continuously, or adequately perceive resistance to insufflation. For this reason, a pressure relief valve must be added to the BVM to ensure performance and safety of the device based on the needs of each patient. The pressure relief valve is not found on all brands of BVM. One must be added if there is none. When connecting the BVM to the device, it must be used with the pressure regulating valve unlocked and the pressure should be monitored with a manometer if one is available [12].

Infection Control

MHRA protocol guided the addition of the following features [3]:

1. All parts coming into contact with the patient’s breath are disposable or designed to be reusable. 2. All working components are contained within an impermeable casing. Healthcare workers are able to manually wipe clean all external surfaces. 3. The AIR can connect to a viral hygroscopic filter.

Operational considerations

The AIR must be used by a professional who is familiar with the characteristics of BVMs and the factors that impact ventilatory outcomes with these devices. In the event of power failure or component breakdown, the self-inflated bag can be easily removed from the AIR so that ventilation can continue manually. In this case, AIR will function similar to an unmodified BVM, although we have added safety mechanisms to AIR following protocols established by the MHRA [3]. The successful operation of AIR, therefore, incorporates two sets of skills:

1. Handling the BVM 2. Operation of AIR

*Handling the BVM* 

AIR’s self-inflated bag, or BVM, can be used automatically or manually. This bag is made of a malleable material and must expand after each compression. The frequency of re-inflation determines the maximum minute volume, which in turn impacts the expiratory flow rate and thus the inspiratory:expiratory (I:E) ratio [13]. As mentioned, the use of the oxygen reservoir will impact the FiO2 offered [6]. In addition, FiO2 can be modified with Venturi valves, which can be printed on 3D printers [10]. Operators should be able to manipulate FiO2 using both of these components.

Operation of AIR

AIR should be used only by qualified and trained professionals. For optimal use, training scenarios should prioritize both technical skill related to the device itself, and nontechnical skill related to the environment in which the device will be used. These skills can be organized into four categories: safety, operation, crisis resource management, and communication.

1. Safety: Proper knowledge and use of Personal Protective Equipment (PPE) must be demonstrated at all times. Also, the AIR non-rebreathing valve can become contaminated with patient fluids during ventilation. For this reason, it is necessary to frequently check the equipment throughout ventilation. Other infection and contamination protocols must be adequately and regularly observed, and the participant should demonstrate sufficient functional knowledge to use AIR safely in each of its operative settings.

2. Operation: The participant should gain a thorough understanding of the AIR’s operative range and capacity. This understanding should be accompanied by technical skill, exhibited by the ability to quickly choose, interconvert, and modify operating modes in response to changing patient conditions. This operative competence is essential for patient safety. Inaccurate compressions of the BVM bag can cause hypoventilation, hyperventilation, barotrauma, and reduced cardiac output. Therefore, the same logic must be followed for AIR and similar devices.

3. Crisis resource management: Since the AIR is intended for limited-resource, high-acuity environments, participants should engage in scenarios that simulate these conditions. Participants should be able to assess environmental cues, identify possible constraints, and prioritize action accordingly. Successful assessment of this measure will require that participants demonstrate situational awareness, which can be divided into three stages: perception (recognizing basic features and dynamics of scenario); comprehension (integrating perceptions into holistic image and understanding relationship to goals); and projection (extrapolating information from first two stages to determine future status of situation) [14].

4. Communication: Communication is an important component in patient outcomes, and high-fidelity simulations have been identified as a method of promoting communication skills relevant to emergency procedures [15,16]. Training scenarios should therefore emphasize communication between and within healthcare teams. Successful assessment of this measure will require that participants demonstrate effective communication in the following three stages: information (accurately exchanging information using a technique such as ‘read back, answer back’); understanding (constructing an acceptable, shared picture of the situation based on information); and coordination (working with others in synchronous, collaborative manner based on understanding) [17]. Communication with the patient and their family is also essential, particularly (in the context of COVID-19) as a method of contact tracing.

Simulation scenario

Prescenario

The following is presented to the participants as a stem of the simulation case:

“You are part of an emergency room team near Tobermory, Ontario, Canada where there is only one doctor and one nurse available. You are called at 7:45 AM to see a 65-year-old man brought in by paramedics with severe respiratory failure. A laryngeal mask airway (LMA) is in place and he is connected to cardiac monitors with a complete set of vital signs. The prehospital team installed hygroscopic filters in the tubes and the BVM, but the risk of the viral propagation remains great. There is only one traditional mechanical ventilator in the hospital and it is already being used by a 40-year-old female patient with severe respiratory failure but not COVID-19. Referral hospitals are not receiving transfers due to the high influx of COVID-19 patients.”

The patient’s history is reported by his 30-year old son, who is waiting in hospital reception with his five-year-old daughter (the patient’s granddaughter). Seven days ago the patient started a dry cough with a fever. The dry cough worsened in the last two days, becoming productive and associated with severe shortness of breath. Earlier this morning he was found in his bed, unconscious and breathing heavily.

Ten days ago, the patient’s son and granddaughter returned from Italy. On the evening of their return they visited the patient for dinner. Today the child has a mild runny nose. The patient’s son had a mild cold three days ago and is feeling well today.

In this scenario, three individuals are infected by SARS-CoV-2 (the virus that causes COVID-19) and the entire simulation will be based on the necessary care relevant to this information. The main areas of focus are safety, including the proper use of PPE; operating AIR when there is no mechanical ventilator available; crisis resource management; and effective communication during a high-acuity clinical event. These learning objectives are displayed in Table 2.

**Table 2:** Learning objectives PPE: personal protective equipment; AIR: automated inflating resuscitator; BVM: bag valve mask.

Learning objectives
1. Safety: Participants should be able to properly select and use, or describe the selection and use of all relevant PPE prior to interaction with the patient and AIR.
2. Operations: Participants should gain sufficient technical skill in operating the AIR and BVM, such that respective functions can be quickly selected, interconverted, and modified in response to changing patient conditions.
3. Crisis resource management: Participants should demonstrate situational awareness of low-resource setting by quickly assessing environmental features, identifying possible constraints, and prioritizing action accordingly.
4. Communication: Participants should exchange information with others to build understanding and coordinate collaboration. Communication should be calm, clear, and concise.

Inputs

Equipment

This simulation can be performed in a controlled environment using a manikin with realistic lungs or an actor. Respiratory parameters should be adjusted to simulate a critical COVID-19 patient. These parameters must be measurable from the point of view of being stabilized by our AIR for a period of time determined by the facilitators. Benchtop simulators can be used for the development of relevant psychomotor/technical clinical skills, such as airway management with the insertion of oropharyngeal airways (OPA), endotracheal intubation, insertion of the chest tube, and other procedures.

This simulation is intended for healthcare professionals operating in a ventilation shortage, who will have to use AIR or similar devices. Therefore, other devices can be used if their basic operative function is sufficiently similar to AIR. Facilitators should make this determination ahead of time in order to modify relevant portions of the scenario and also the assessment of operative skill (Table 2: Learning Objective 2) as required. This simulation will be performed in a rural or remote hospital environment with few resources. The recommended materials, drugs, and equipment are listed in Appendix 2. 

Information

**Figure 3:**
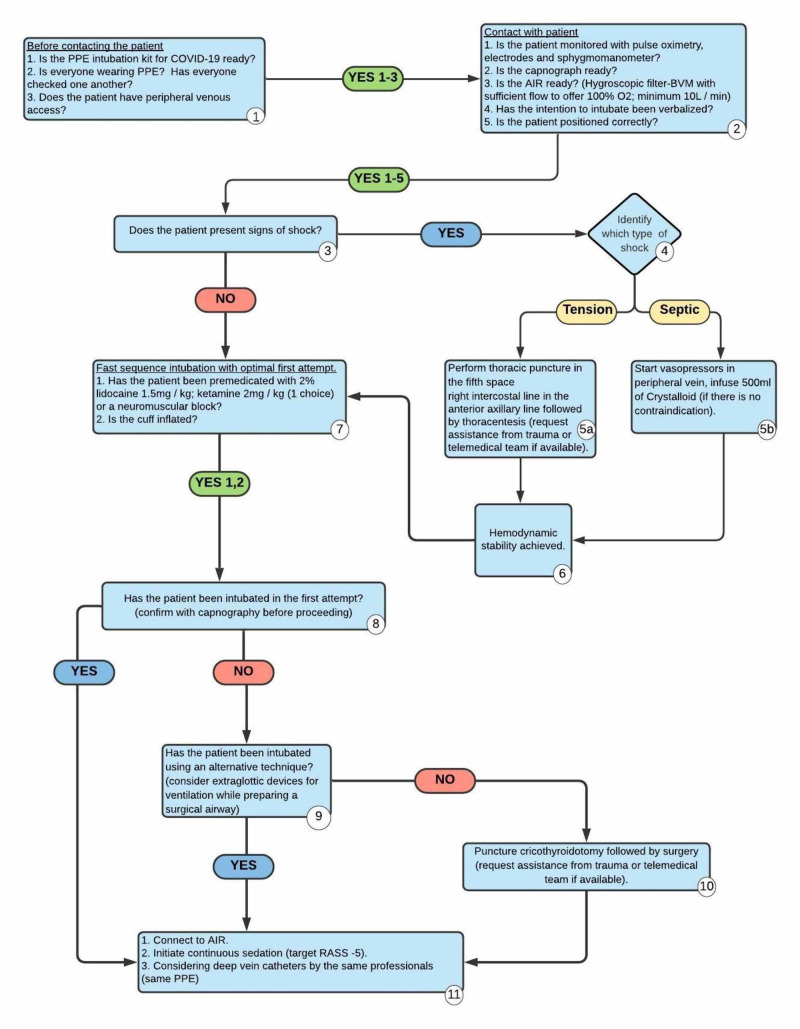
Intubation procedure sequence PPE: personal protective equipment; AIR: automated inflating resuscitator; BVM: bag valve mask; RASS: Richmond Agitation-Sedation scale.

Upon request, participants receive a list of patient data, including allergies, medications, and medical history (see Appendices 3 and 4). The facilitators will be also given a list of the sequence to be followed in the patient's intubation procedure (Figure 3). The facilitators choose the best path to be taken by the participants.

Ideally, the simulation is intended for healthcare professionals working in emergency care in rural areas or seeking additional training in the treatment of patients with severe respiratory failure. As the simulation takes place in a hospital environment, there is access to images and laboratory investigations. However, these resources may be limited or not. This will be at the facilitators' discretion and in accordance with local capacity. Further, the facilitators should decide which regional, national, or international protocols will be observed. Since these guidelines are currently being developed, we will not adopt any in particular.

Facilitators

Two healthcare professionals, both of whom are comfortable caring for patients with severe respiratory failure, should act as facilitators. These professionals should have experience with the use of traditional ventilators and manual resuscitators. One should be designated as the primary facilitator, providing guidance to the participants and helping with the general organization and execution of the case, while the second facilitator will be present to assess individual performance. Facilitators are responsible for providing participants with the appropriate information as requested, including vital signs and other relevant physical examination results (see Appendices 3 and 4). Facilitators should examine the scenario in advance to identify possible limitations or technical problems, and they should also take time to fully familiarize themselves with AIR’s functionality.

Context

The participants start the case in an emergency room (ER), which contains a doctor and a nurse (the number of professionals can be changed). The only traditional mechanical ventilator is already in use by a non-COVID-19 patient, who has required ventilatory support since last night. This small hospital is 250 km from Toronto where, despite a far greater number of intensive care unit (ICU) beds with ventilators, there is a long list of patients who also need this equipment. The Toronto hospital informs your staff that it is unable to receive new critically ill patients. However, the local team has access to a mechanical ventilator that was developed from a BVM. This is the only available device capable of keeping a patient with severe respiratory distress alive for a prolonged period of time. In this simulation case, we presented three people that are infected by SARS-CoV-2. A 65-year-old elderly man with severe respiratory failure, an asymptomatic 30-year-old adult man, and a 5-year-old child with only a mild runny nose (these last two are in the waiting room).

Before starting the simulation, participants must be submitted to the fictional contract, which recognizes that all processes occurring during the simulation should be treated as if they were "real", so that the objectives of the simulation can be achieved. During this period, facilitators present the simulation scenario and all necessary precautions, before introducing the AIR and describing its function. Finally, participants are encouraged to assign roles in preparation to receive the patient. If there is a limited supply of participants, the paramedics team can only be reported by the facilitators, as the main objective of this simulation is training with the use of AIR and care involving a patient with COVID-19. Figure 3 displays the sequence to be followed in the patient's intubation procedure.

Table 3 shows the objectives of the simulation in detail. The scenario begins with the patient in bed and the participants outside of the room.

**Table 3:** Clinical scenario checklist PPE: personal protective equipment; AIR: automated inflating resuscitator; BVM: bag valve mask; RASS: Richmond Agitation-Sedation scale; ICU: intensive care unit.

Objectives checklist		
Objective 1. Safety: Participants should be able to properly select and use, or describe the selection and use of all relevant PPE prior to interaction with the patient and AIR.		
Expected action:	Findings/Justification:	Completed (Y/N):
Offer surgical masks for all people with respiratory symptoms when entering the hospital.	Findings: The patient's son and granddaughter are without masks.	
	Justification: They can be contaminated and contaminate people nearby.	
Guide the correct use of the surgical mask for those with respiratory symptoms (cover mouth and nose).	Findings: The patient's son and granddaughter are having trouble using the mask correctly.	
	Justification: Misuse of the mask can contaminate people nearby.	
Determine the availability of room used for infection control (e.g., negative pressure room). If none is available, adjust airflow through receiving room to minimize risk of airborne transmission. Make seasonal modifications as necessary.	Findings: In the summer: the window is closed, the fan is on and the door is open. In winter: the draft heater is on.	
	Justification: The air stream can carry aerosols to other rooms and contaminate nearby environments.	
Confirm that the team has properly disinfected the room and instruments used (including AIR) before and after patient visits with 70% alcohol or 5% sodium hypochlorite.	Findings: Participants did not ensure the room, instruments, and AIR were adequately cleaned before receiving the patient.	
	Justification: Failure to adequately and regularly clean surfaces before and after patient visits increases infection risk.	
Wash hands with water and liquid soap or use 70% alcohol before using PPE.	Findings: Participants wear PPE without washing their hands with the correct technique.	
	Justification: Contamination risk.	
Use closed shoes, mask, waterproof apron, glasses, gloves, and face shield (when in contact with aerosols).	Findings: Participants do not wear all PPE.	
	Justification: Contamination risk.	
Avoid touching eyes, nose, and mouth.	Findings: Participants touch their eyes, face, mouth, or hair.	
	Justification: Contamination risk.	
Ensure that everyone is properly wearing PPE, and ask others to ensure their own PPE is properly worn.	Findings: Participants do not check that their colleagues are properly wearing PPE and do not ask colleagues to check their own PPE.	
	Justification: Improper or incomplete use of PPE leads to a high risk of contamination for all team members.	
Remove personal ornaments (rings, bracelets, watches, necklaces, earrings, etc.).	Findings: Participants are wearing watches, earrings, necklaces, bracelets and etc.	
	Justification: There is a risk of contamination of ornaments and a great risk of contaminating people	
Remove the mask by pulling on the elastic or strips, taking care that there is no contact with the external surface, in the same way with gloves, aprons and other PPE when finalizing patient care.	Findings: When withdrawing PPE the participants do not follow the correct aseptic withdrawal protocol.	
	Justification: Contamination risk.	
Discard disposable materials in the infectious waste.	Findings: Participants discard PPE in non-designated receptacles, such as common garbage.	
	Justification: High risk of contamination of the environment and people.	
Objective 2. Operations:Participants should gain sufficient technical skill in operating the AIR and BVM, such that respective functions can be quickly selected, interconverted, and modified in response to changing patient conditions.		
Expected action:	Findings/outcome:	Completed (Y/N):
Recognize that supraglottic device (laryngeal mask) must be exchanged for a definitive airway device such as endotracheal tube or cricothyroidotomy (Figure 3).	Findings: Participants have not demonstrated knowledge that a supraglottic device should be exchanged for a definitive airway.	
	Justification: Lesions of the mucosa of the uvula and posterior wall of the hypopharynx, compressions of vessels, evolving with cyanosis of the tongue and macroglossia for a few hours, compression of the lingual nerve, with anesthesia in topography of the path of this nerve, compression of the recurrent laryngeal nerve and, consequently, uni or bilateral vocal fold paralysis, leading to dysphonia and compression of the hypoglossal nerve, with transient paralysis of tongue movements.	
Verbalize intention to exchange the laryngeal mask for a definitive airway (endotracheal tube or cricothyroidotomy) (Figure 3).	Findings: Participants do not verbalize their intention to change the non-definitive airway to a definitive one.	
	Justification: Lack of coordination among team members can lead to loss of time in requesting the necessary equipment for this procedure.	
Pre-oxygenate the patient using the BVM with sufficient flow to guarantee O2 to 100% (minimum 10 L/min) (Figure 3).	Findings: Participants perform inaccurate compressions (either too strong or too weak), make sudden movements in the BVM and the tube and do not adjust an oxygen flow greater than or equal to 10 L/min.	
	Justification: 1. If very strong: barotrauma and pneumothorax. 2. If very weak: little oxygen supply and worsening the clinical picture. 3. Sudden movements of the BVM and tubes: laryngeal mask moves and the patient loses the airway. 4. If oxygen flow less than 10 L/min: little oxygen offered and worsening of the clinical picture.	
Position the patient appropriately to start exchanging the laryngeal mask with an orotracheal tube and the participant is positioned behind the patient's head (Figure 3).	Findings: The participants do not position the patient to begin the intubation procedure and the participant is not positioned behind the patient's head.	
	Justification: The intubation will be compromised, the patient may worsen the clinical condition and there is an increased risk of contamination of the team.	
The patient must be adequately sedated and have no airway complications. Fast sequence intubation with the first optimal attempt: Removed supraglottic device and performed intubation by endotracheal cannula and inflated the cuff (Figure 3).	Findings: The participants start with the first of drugs. After failure with the first choice, participants go to the second choice. After initiating the second choice of drugs, the patient is adequately sedated and the exchange of the supraglottic device for the orotracheal cannula was successfully performed (Figure 3).	
	Justification: Facilitators should guide this sequence to teach participants that the patient must be adequately sedated in order to exchange the supraglottic device for an endotracheal cannula.	
Request the use of capnography to confirm orotracheal intubation, especially in the context of difficult visualization caused by the use of PPE (Figure 3).	Findings: Participants use the stethoscope instead of a capinographer to verify that the orotracheal tube was installed properly.	
	Justification: Contamination risk..	
Connect to the BVM and properly ventilate the patient (Figure 3).	Findings: Participants choose the wrong Venturi valve, perform inaccurate compressions of the bag (either too strong or too weak), make sudden movements in the BVM and the tube, or do not adjust an oxygen flow greater than or equal to 10 L/min.	
	Justification: 1. If very strong: barotrauma and pneumothorax. 2. If very weak: little oxygen supply and patient worsens the clinical picture. 3. Sudden movements of the BVM and tubes: laryngeal mask moves and the patient loses the airway. 4. If oxygen flow less than 10 L/min: little oxygen offered and worsening of the clinical picture.	
Adjust AIR parameters prior to patient connection (Table 1).	Findings: Participants do not adjust AIR parameters before connecting patient.	
	Justification: Time is wasted adjusting AIR parameters after patient is connected.	
Transfer patient from BVM to AIR using a Kelly clamp (Figure 3).	Findings: Participants do not clamp the tube before switching from BVM to AIR.	
	Justification: A Kelly clamp is important for clamping the tube when there is a need to change circuits/ventilators, in order to minimize aerosol production (Figure 3).	
Initiate continuous sedation. Target RASS: -5 (Figure 3).	Findings: Participants do not initiate continuous sedation.	
	Justification: Patient "struggles" with the ventilator and/or has a cough reflex and worsens the clinical picture.	
Objective 3. Crisis resource management: Participants should demonstrate situational awareness of low-resource setting by quickly assessing environmental features, identifying possible constraints, and prioritizing action accordingly.		
Expected action:	Findings/outcome:	Completed (Y/N):
Do not waste PPE.	Findings: Participants waste gloves, masks, etc.	
	Justification: Resources are already limited and wasting PPE can lead to huge losses.	
Ration equipment and medications appropriately.	Findings: Participants waste tubes, drugs, etc.	
	Justification: Resources are already limited and wasting equipment and medications can lead to huge losses.	
Request laboratory/imaging tests only when required (see Appendix 5).	Findings: Participants order laboratory or imaging tests when they are not necessary.	
	Justification: Wasting laboratory/imaging tests will prevent these resources from helping patients who need them.	
Collect information about medical care limitations from the referral hospital (number of beds in the ICU and number of mechanical ventilators).	Findings: Participants do not collect information on the number of beds in ICUs and the number of mechanical ventilators.	
	Justification: The lack of knowledge about the care capacity of reference hospitals and the number of mechanical ventilators can lead to great human losses and ineffective care for new patients.	
Order modern mechanical ventilator or transfer patient to nearest available ICU.	Findings: The participants do not request a modern mechanical ventilator to exchange for AIR and do not request a transfer to an ICU	
	Justification: The patient’s clinical condition can worsen if he is not connected to a modern mechanical ventilator and/or he is not transferred to an ICU.	
Objective 4. Communication: Participants should exchange information with others to build understanding and coordinate collaboration. Communication should be calm, clear, and concise.		
Expected action:	Findings/outcome:	Completed (Y/N):
Before patient arrival		
Obtain all relevant patient information from paramedics and/or prehospital team.	Findings: Participants do not collect patient information from paramedics, such as why they were called, how they found the patient, vital signs, who their companions are, where they are and how many there are.	
	Justification: Not collecting information from the prehospital team can lead to poor patient care and a high risk of contamination by the team because they are not prepared to receive a suspect from COVID-19.	
Share patient information with the rest of their team.	Findings: Participants who collected complete patient information do not share it with the rest of the team.	
	Justification: Failure to share information with the rest of the team can put patient care at risk and also a high risk of contamination by the team.	
Clearly and confidently communicate a plan based on patient information.	Findings: Participants neglect the importance of the correct use of PPE, the importance of exchanging a non-definitive airway for a definitive one, the importance of requesting a mechanical ventilator for long-term use and / or transfer to an ICU and the importance of an effective communication to gather all information about the lack of available resources.	
	Justification: Not understanding all stages of: care to avoid contamination, correct use of AIR and communication with the pre-hospital team and the referral hospital can put the patient's life at risk and a high risk of contamination by the medical team.	
Confirm understanding following communication.	Findings: Participants fail to ensure information has been exchanged fully and accurately.	
	Justification: Participants should employ a strategy such as ‘read back, answer back’ to maintain a shared understanding of the situation, allowing them to develop collaborative action accordingly.	
Patient interaction		
Obtain patient’s medical history.	Findings: Participants do not collect the patient's complete medical history.	
	Justification: Failure to collect a complete patient history can lead to diagnostic errors, ineffective treatment, risk of contamination by the medical team and ineffective isolation conduct with family members suspected of being infected with COVID-19, causing them to contaminate close people.	
Make an effort to establish trust and rapport with the patient's family.	Findings: Participants do not share all the information about the patient with family members, for example: the diagnostic hypothesis, their clinical condition, the treatments proposed in a situation where resources are limited and about the great efforts of the medical team to refer the patient to the best possible destination such as an ICU.	
	Justification: The lack of communication with family members can put the doctor-patient relationship at risk.	
Patient transfer		
Confirm understanding following communication.	Findings: Participants do not inform or collect vital information about the patient with paramedics and/or family members or between the team. Participants do not share valuable patient information with the referral hospital.	
	Justification: The lack of communication between pre-hospital staff, family members and reference hospital can put the patient's life at risk and a high degree of contamination among all involved.	
Share all relevant patient information with the referral hospital and keep up to date on the availability of an ICU bed.	Findings: Participants do not share all the details of the patient with the referral hospital, such as: diagnostic hypothesis, ongoing treatment, requested laboratory and imaging tests and isolation conduct with close family members. participants also showed no interest in constantly updating themselves on the availability of an ICU bed to transfer the patient or not updating themselves on the availability of a modern mechanical ventilator.	
	Justification: Failure to share all information about the patient with the referral hospital can put the patient's life at risk and/or high risk of contamination by the medical staff of the hospital that will receive the patient. Failure to update about the availability of an ICU bed and a mechanical ventilator can put the patient's life at risk.	

Debriefing

During this session, the “debriefing with good judgement” model can be used [18]. This model uses an advocacy-inquiry approach to initiate a process of self-reflection, frame analysis, and behavioural change. In this method, facilitators address unexpected actions by first indicating their own expectation regarding correct action (advocacy), and then testing that expectation by investigating the cognitive frame that led to participant action (inquiry). The rationale behind this strategy is to clearly identify discrepancies between expected and performed actions without eliciting a defensive or otherwise counterproductive response on the part of the learner [18]. In this way, the facilitator attempts to cultivate favourable conditions for self-reflection. Participants should have the opportunity to share their positive and negative experiences, and facilitators should invite suggestions on how the simulation can be improved.

This can be followed by a didactic session in which facilitators demonstrate appropriate use of PPE, as well as operative functions of the AIR and BVM. Facilitators can also take this time to share and discuss strategies for nontechnical skills relevant to crisis resource management and team communication. Lastly, facilitators may provide details relevant to the medical regulation of communication between institutions and local ambulance teams.

## Discussion

The aim of this technical report was twofold: first, to describe the design and manufacturing process of the AIR, a 3D-printed ventilator training device which operates on the principle of pushing a BVM; second, to describe a simulation scenario that can be used for training health professionals how to effectively and safely use this and similar, low-cost, 3D-printed ventilators in the context of ventilator shortages caused by COVID-19.

The development and manufacturing of the ventilator followed an iterative design cycle between clinical experts (ER doctors), mechanical engineers, and education researchers. The team used MHRA guidelines to develop the initial ventilator. The development and manufacturing took four weeks, with equipment costs of $100 CAD, and human resource commitments: Doctors - 130 hours, engineers - 100 hours, researchers - 100 hours. Proper assessment of the clinical functioning of this prototype is outside of the scope of this technical report, and will be presented in a follow-up case study.

Based on the recommendation of Scalese et al. describing the effectiveness of simulation training for high-acuity clinical environments, we have also developed a simulation scenario [19]. Our scenario helps front line health professionals become familiar with the use of AIR in terms of safety and operations. Further, the scenario was aimed to train crisis resource management and communication skills when operating AIR in the context of a ventilation shortage. While the scenario is based on COVID-19, it can be adapted to various settings where access to care and resources may be limited.

Our scenario also emphasizes the care of every patient who needs ventilatory support, not only those caused by COVID-19, as the deficiencies here can significantly impact care for all types of critically-ill patients.

Long-term use

The AIR prototype exhibits most of the minimum MHRA specifications, but additional components should be added prior to clinical use [3]. To this end, the most urgent areas of future research are described below: 

Alarms

Gas or electricity supply failure; Inspiratory airway pressure exceeded; Inspiratory and PEEP pressure not achieved (equivalent to disconnection alarm); TV not achieved or exceeded. 

Monitoring

TV; frequency; PEEP; FiO2; ventilation mode; actual current airway pressure. Monitors should display continuously so the user can verify settings and modify as needed. If pressure support mode is provided there must be real time confirmation of each patient's breath and an alarm if below acceptable range.

## Conclusions

The first aim of this report was to describe the design and manufacturing process of a 3D-printed ventilator training device, the automated inflating resuscitator (AIR), which operates on the principle of pushing a bag valve mask (BVM). The current AIR prototype exhibits many of the MHRA safety requirements, but an assessment of clinical viability falls outside the scope of this report. The modular design does however provide a template upon which further functions can be added. The second aim of this report was to describe a simulation scenario that can be used for training frontline workers in how to use AIR and similar ventilators. The scenario was designed to train safety, operative ability, crisis resource management, and communication skills. The context focused on ventilation shortages caused by COVID-19, but it may be adapted to simulate other conditions where similar ventilation devices are used.

## Appendices

Appendix 1 

**Table 4:** AIR components 3DP: 3D-printed; Dc: Direct current; M: Metric; ISO: International Organization for Standardization

ITEM NO.	PART NUMBER	DESCRIPTION	QTY.
1	BaseLayoutBoard	Fabricated/3DP	1
2	MotorBase	3DP	1
3	Motor	Purchased	1
4	Motor Speed Controller	Purchased	1
5	DcPowerSupply	Purchased	1
6	CamBase	3DP	1
7	MotorShaftCoupling-Bottom	3DP	1
8	MotorShaftCoupling-Top	3DP	1
9	Bearing (Dimensions: 20x42x12 mm)	Purchased	1
10	CamCoupling1	3DP	1
11	CamCoupling2	3DP	1
12	Cam	3DP	1
13	CamPadRailBase	3DP	2
14	CamPadRail	3DP	1
15	CamPadRailShaft1	Fabricated/3DP	1
16	CamPadRailShaftLatch	3DP	1
17	CamPadRailConnector	3DP	1
18	CamContactPad	3DP	1
19	CamPadRailShaft2	Fabricatedd/3DP	1
20	RopePipe	3DP	1
21	RopeExtender	3DP	1
22	BagBase	3DP	1
23	BagRailBar1	Fabricatedd/3DP	1
24	BagKnobBase1	3DP	1
25	BagRailBar2	Fabricatedd/3DP	2
26	BagKnobBase2	3DP	1
27	Rack1	Fabricated/3DP	1
28	Gear	Fabricated/3DP	2
29	Rack2	Fabricated/3DP	2
30	BagRailBar2Latch	3DP	1
31	BagHolderL	3DP	1
32	BagHolderR	3DP	1
33	BagKnob	3DP	2
34	BagBaseCover	Fabricated/3DP	1
35	BagKnobHead	3DP	2
36	SelfInflatedBag	Purchased	1
37	GearLatch	3DP	2
38	MotorCoverBoardL	Fabricated/3DP	1
39	MotorCoverBoardBack	Fabricated/3DP	1
40	MotorCoverBoardFront	Fabricated/3DP	1
41	MotorCoverBoardR	Fabricated/3DP	1
42	MotorCoverBoardTop	Fabricated/3DP	1
43	Rope	Purchased	2
44	ISO 4010 Bolt M5 x 30 mm	Purchased	4
45	Bolt M5 x 40 mm (ISO 4014)	Purchased	8
46	Bolt M5 x 45 mm (ISO 4014)	Purchased	10
47	RubberBand	Fabricated/Purchased	2
48	RubberBand2	Fabricated/Purchased	2
49	Nut M5 (ISO 4034)	Purchased	22
50	Nut M3 (ISO 4034)	Purchased	69
51	Bolt M3 x 16 mm (ISO 4762)	Purchased	6
52	Bolt M3 x 20 mm (ISO 4762)	Purchased	1
53	Bolt M3 x 25 mm (ISO 7045)	Purchased	29
54	Bolt M3 x 12 mm (ISO 7045)	Purchased	1
55	Bolt M3 x 30 mm (ISO 7045)	Purchased	5
56	Bolt M3 x 20 mm (ISO 7045)	Purchased	17
57	Bolt M3 x 16 mm (ISO 7045)	Purchased	4
58	Bolt M3 x 10 mm (ISO 7045)	Purchased	4

 Appendix 2

**Table 5:** Recommended resources PAPR: Powered Air Purifying Respirator; BVM: Bag Valve Mask; AIR: Automated Inflating Resuscitator.

Resource	Description
PPE	1. Waterproof apron and 2 pairs of gloves; 2. N95 mask or PAPR. Surgical mask is acceptable patients not undergoing aerosol-generating procedures. An anesthesia mask might be placed in-line with a viral filter to MacGyver an N95 mask; 3. Goggles or face shield; 4. Hair cover for aerosol-generating procedures; 5. Hood may be used, especially during intubations. This can be constructed out of a trash bag; 6. Shoes: Shoes that are easily cleaned and don't need to be touched are preferred. Shoe covers aren't recommended as removing them may increase exposure.
Material	1. Bougie and guide wire, Videolaryngoscope (disposable blades number 3 and 4) (if available); 2. Conventional laryngoscope (curved blades numbers 3 and 4, straight blade number 4); 3. Orotracheal tube, numbers 7.0, 7.5, 8.0, and 8.5; 4. Scalpel number 22 plus tracheostomy cannula number 4.5 or orotracheal tube number 5.0 with cuff or own puncture cricothyroidotomy kit; 5. Hygroscopic filter (to be used in the BVM and ventilator circuit); 6. Extraglottic device (Laryngeal Mask or Laryngeal Tube, number 3, 4, and 5); 7. Straight Tweezers - Kosher or Curve - Kelly Camp; 8. Stethoscope; 9. Portable ultrasound (if available). 10. Single-use end tidal CO2 detector.
Drugs	1. Rocuronium: 10 mg/mL; 2. Succinylcholine: 100 mg powder for solution for injection; 3. Ketamine: 50 mg/mL; 4. Lidocaine: 2% without vasoconstrictor; 5. Epinephrine: 1 mg/mL; 6. 0.9% NaCl solution or Ringer Lactate 500mL; 7. Norepinephrine: 2mg/mL.
Equipment	1. Mechanical ventilation circuits (BVM and AIR); 2. Monitor & capnograph (if available); 3. Infusion pump with 3 channels or 3 infusion pumps; 4. For peripheral access: 1 pair of procedure gloves, alcohol swab, micropore 20cm, scissors, tourniquet, peripheral venous catheter, two-way connection, tray; 5. For central access: single, double or triple lumen catheter, central access puncture tray, chlorhexidine degermante, alcoholic chlorhexidine, sterile apron, gloves, mask, cap, glasses, micropore tape; 5 mL and 10 mL syringes; 40x12 and 30x7 or 25x7 needles, 2% xylocaine without vasoconstrictor, suture thread (3-0 nylon), scalpel blade number 11, sterile gauze, cardiac monitor (if available).

Appendix 3

**Table 6:** Patient history BVM: Bag Valve Mask.

Category	Information
History of presenting illness	Seven days ago the patient started with a dry cough associated with headache and fever of 39.5°C. He used paracetamol, which improved headache and lowered fever to 37.2°C for one day and returning to 39-40°C the next day. The dry cough became productive 2 days ago associated with severe dyspnea and a fever that did not go down with the medication. This morning his son tried to call him but unsuccessfully. Going to his house he found him unconscious and breathing hard in his bed. The patient was admitted by paramedics, under positive pressure ventilation, using a laryngeal mask and using a BVM with filters to contain virus.
Allergies	none
Medications	none
Medical History	Appendectomy at age 12. Bacterial pneumonia at age 60, treated with azithromycin and recovered fully.
Lifestyle	Has never smoked or drank alcohol. Swims every day and has eaten well for years.
Family History	Father died in a car accident 60 years ago and his mother died 2 years ago of natural causes. No history of diseases. No siblings.

 Appendix 4

**Table 7:** Patient physical exam T: Temperature; HR: Heart rate; BP: Blood pressure; RR: Respiratory rate; SpO2: Oxygen saturation; FiO2: Fraction of inspired oxygen.

Category	Information
General	Patient in critical condition in a deep coma, ventilated with supraglottic device
Initial Vitals	T: 40.1 °C axillary; HR: 110; BP: 130/80; RR: under compression bag; SpO2: 71%; FiO2: 100%.
Head, Eyes, Ears, Nose, Throat	Nothing
Central Nervous System	Glasgow Coma Scale 3 and no vomit reflex.
Chest	No distended neck veins, no tracheal deviation, right chest with diminished breath sounds, hyperresonance with percussion in aplex and middle third, discrete asymmetric chest wall excursion (decreased excursion on the right). Left side normal.
Cardiovascular System	Normophonetic sounds, in two stages, without murmurs. No jugular vein distension
Abdomen	Scar located in McBurney region compatible with surgical history. Soft and non-tender, bowel sounds present.

Appendix 5 

**Table 8:** Laboratory and imaging tests RT-PCR: Reverse transcription polymerase chain reaction; SARS-CoV-2: Severe acute respiratory syndrome coronavirus 2; CT: Computed tomography; PT: Prothrombin time; PPT: Partial thromboplastin time.

Number	Type
1	RT-PCR in real time
2	SARS-CoV-2 rapid molecular test
3	Partial or total sequencing of the viral genome
4	Serological tests
5	Blood culture to rule out other causes of infection from lower respiratory tract
6	Arterial blood gases (assess for the presence of hypercarbia or acidosis.)
7	Chest CT
8	Glucose
9	Urea
10	Total bilirubin and fractions
11	D-dimer
12	Complete blood count
13	Coagulogram (PT and PPT)
14	Inflammatory markers (serum procalcitonin and/or Creative protein, depending on availability)
15	Serum troponin; and serum lactate dehydrogenase
